# Oral Health-Related Knowledge, Attitudes and Behaviours of Arab Dental Students: Multi-National Cross-Sectional Study and Literature Analysis 2000–2020

**DOI:** 10.3390/ijerph19031658

**Published:** 2022-01-31

**Authors:** Abanoub Riad, Nuraldeen Maher Al-Khanati, Julien Issa, Mazen Zenati, Nèziha Ben Abdesslem, Sameh Attia, Martin Krsek

**Affiliations:** 1 Department of Public Health, Faculty of Medicine, Masaryk University, 62500 Brno, Czech Republic; krsek@med.muni.cz; 2 Department of Oral and Maxillofacial Surgery, Faculty of Dentistry, Syrian Private University, Damascus 368, Syria; nuraldeen.alkhanati@gmail.com (N.M.A.-K.); m-zenati@hotmail.com (M.Z.); 3 Department of Oral and Maxillofacial Surgery, Faculty of Dental Medicine, Damascus University, Damascus 222, Syria; 4 Faculty of Dentistry, Beirut Arab University, Tarik El Jadida, Beirut 11-50-20, Lebanon; julien21issa@gmail.com; 5 Department of Biomaterials and Experimental Dentistry, Poznań University of Medical Sciences, 60-781 Poznan, Poland; 6 Faculty of Dental Medicine, University of Monastir, Monastir 5019, Tunisia; neziha.ben.abdesslem@gmail.com; 7 Department of Oral and Maxillofacial Surgery, Justus-Liebig-University, Klinikstrasse 33, 35392 Giessen, Germany; sameh.attia@dentist.med.uni-giessen.de

**Keywords:** Arab Countries, dental education, dental students, health knowledge, attitudes, practice, Hiroshima University Dental Behavioural Inventory—HU-DBI, Lebanon, oral health, oral hygiene, Syria, Tunisia

## Abstract

Dental students are the future leaders of oral health in their respective communities; therefore, their oral health-related attitudes and behaviours are of practical value for primary disease prevention. The present study aimed to evaluate oral health-related knowledge, attitudes, and behaviours of dental students in Arab countries and explore the potential sociodemographic predictors of their oral health outcomes. A multi-centre, cross-sectional study was conducted during the academic year 2019/2020 in three Arab countries: Lebanon, Syria, and Tunisia. The study used a validated Arabic version of the Hiroshima University Dental Behavioural Inventory (HU-DBI) composed of original twenty items that assess the level of oral health-related knowledge, attitudes, and behaviours, and four additional dichotomous items related to tobacco smoking, alcohol drinking, problematic internet use, and regular dental check-up The HU-DBI score ranges between 0 and 12. A total of 1430 students took part in this study, out of which 60.8% were females, 57.8% were enrolled in clinical years, 24.5% were tobacco smokers, 7.2% were alcohol drinkers, and 87% reported internet addiction. The mean HU-DBI score was 6.31 ± 1.84, with Lebanon having the highest score (6.67 ± 1.83), followed by Syria (6.38 ± 1.83) and Tunisia (6.05 ± 1.83). Clinical students (6.78 ± 1.70) had higher HU-DBI scores than their preclinical peers (5.97 ± 1.86). The year-over-year analysis revealed that dental public health and preventive dentistry courses had significantly and positively impacted the undergraduate students’ knowledge, attitudes, and behaviours. The gender-based differences were not statistically significant, with a modest trend favouring males, especially oral health behaviours. Tobacco smoking, alcohol drinking, and problematic internet use were associated with lower HU-DBI scores. In the Arab world, the economic rank of the country where the dental students live/study was weakly correlated with the students’ mean HU-DBI score.

## 1. Introduction

In the last thirty years, the significant shift of the global burden of disease (GBD) towards non-communicable diseases (NCDs) has drawn the attention of the international community, represented by the World Health Organization (WHO) [1,2]. As the United Nations (UN) recognises NCDs as a major challenge for the sustainable development goals (SDGs) agenda, the WHO developed a global coordination mechanism for the prevention and control of NCDs that aims to reduce NCDs-related premature mortality by one-third by 2030 [1,3]. Oral diseases are the most prevalent NCDs globally, which affect people of all genders, races, age groups and socioeconomic levels; furthermore, to explain the global burden of oral diseases, it is worthy to note that one out of every two adult humans suffers from untreated dental caries [4].

Modifiable risk factors related to lifestyle choices represent the largest portion of the underlying aetiology of NCDs; therefore, public health programs aim to first control them [5,6,7]. The common risk factor approach (CRFA) that Sheiham and Watt proposed in 2000 is based on the notion that oral diseases are multifactorial and can respond strongly to the interventions that target oral hygiene habits, diet, smoking, stress coping mechanisms, and patterns of seeking professional care [8,9,10].

Healthcare professionals play a central role in shaping their patients’ health-related attitudes and behaviours because they are widely perceived as role models of a healthy lifestyle [11]. General physicians’ positive health-related beliefs and behaviours increase their preparedness and capacity to counsel patients on behavioural changes like smoking cessation, using seat belts and reducing fat intake [12]. Therefore, self-care is a core competence of medical education and cost-effective public health intervention for sustainable health promotion [13]. Dental students are the future opinion leaders of oral health in their communities, and their oral health attitudes reflect both their level of understanding of the value of disease control and their role in the primary prevention of oral diseases [14]. Therefore, oral health behaviours of dentists and dental hygienists may act as examples to be followed by their patients, families and friends [15].

The curricula of dental schools can influence students’ oral health attitudes and behaviours while they proceed with their studies. In this context, oral health promotion has been evaluated in various dental curricula by measuring dental students’ clinical outcomes and health attitudes. The current body of evidence suggests that clinical students have better oral health attitudes than their preclinical peers in several countries, e.g., Croatia, Peru and Turkey [16,17,18]. On the contrary, other studies, such as those which were carried out in Yemen and India, did not find any significant correlation between the study level and oral health attitudes and behaviours of dental students, thus suggesting urgent curricular amendments to introduce/increase the preventive dentistry component [19,20].

A recent systematic review for gender differences in preventive behaviours concluded that females were more health-conscious and adopted more preventive behaviours than their male counterparts in all sub-types of primary prevention, including dental hygiene and nutrition [21]. Gender disparities in oral health can be contradicted by the professional knowledge acquired by dental students during their undergraduate education. For example, in Finland, Iran and Japan, gender differences among dental students were absent in cross-sectional studies [15,22]. Nevertheless, a longitudinal meta-analysis observed gender-specific differences among Greek dental students [23].

The common risk-factor approach addresses risk behaviours common to multiple non-communicable diseases, thus providing a solid rationale for improving general health through promoting good oral health [2,8]. Aflalo et al. 2018 found that positive general health behaviours and attitudes were associated with better oral health behaviours in a dose–response association [24]. The same relationship was found in several populations, e.g., Sweden [25,26,27]. Physical activity and smoking are significantly associated with oral health habits in UAE, Finland and ASEAN countries [28,29,30]. Problematic internet use is a common psychosocial phenomenon among adolescents and young adults that directly impacts sleep quality and indirectly affects oral health behaviours [31].

The Arab world is broadly understood as the twenty-two member states of the Arab League whose official language is Arabic and who share sociodemographic and cultural similarities in addition to their geographic proximity [32,33,34]. Nevertheless, the use of this term in public health research has been recently criticised because it incorporates countries of heterogonous economic and political capacities [32]. Therefore, the classification of the World Bank, which is based on gross national income (GNI) per capita, is by far the best approach to classify those countries according to their economic development, which may predict health system integrity and functionality [34,35,36,37]. According to the World Bank, Arab countries belong to the four strata of income as Bahrain, Kuwait, Oman, Qatar, Saudi Arabia, and the United Arab Emirates are high-income countries, while Iraq, Jordan, Lebanon, and Libya are upper-middle-income countries, Algeria, Comoros, Djibouti, Egypt, Mauritania, Morocco, Palestine, and Tunisia are lower-middle-income countries, and Somalia, Sudan, Syria, and Yemen are low-income countries [35].

Despite the recent developments of oral health services in the Arab world, oral health systems in the region rely primarily on out-of-pocket expenditures that create socioeconomic disparities in terms of oral healthcare accessibility [38]. Consequently, a significant rise of oral diseases and their related complications has been consistently reported in the Arab world throughout the last three decades [39]. Therefore, coverage of preventive and restorative services and multi-sectoral approaches utilising epidemiological data are strongly recommended for better control of oral diseases, especially dental caries and periodontal disease [34,39].

The Hiroshima University-Dental Behavioural Inventory (HU-DBI) of Kawamura has been frequently used to assess university students’ oral health-related knowledge, attitudes, and behaviours due to its high psychometric properties that associate students’ replies with clinical outcomes, including dental caries and periodontal diseases [40,41,42]. During the last 30 years, the HU-DBI has been used by dental researchers in more than 10 European countries, including Belgium, Croatia, Finland, France, Germany, Greece, Italy, Lithuania, Turkey, and the United Kingdom [43,44,45,46]. The use of standardised psychometric instruments such as the the HU-DBI is a prerequisite to conducting multi-centre studies that aim to evaluate the self-reported outcomes of populations from different socioeconomic backgrounds [43,44].

This study aimed to evaluate oral health-related knowledge, attitudes and behaviours among dental students in Arab countries. The primary objectives were: (i) to measure the levels of oral health-related knowledge, attitudes, and behaviours among dental students in Lebanon, Syria, and Tunisia using HU-DBI, and (ii) to explore the associations between oral health outcomes of the target population and social determinants of health, e.g., gender, academic level, and clinical training, and their other general health behaviours. The secondary objective was to review the pre-existing body of evidence on oral health-related knowledge, attitudes, and behaviours of Arab dental students assessed by HU-DBI.

## 2. Materials and Methods

### 2.1. Design

The first part of this study had been designed as a multi-centre analytical cross-sectional survey-based study that utilised a digital self-administered questionnaire (SAQ) to collect data from dental students in three Arab-speaking countries. The STrengthening the Reporting of OBservational studies in Epidemiology (STROBE) guidelines for cross-sectional studies had been used to guide the design, conduction, and reporting of this part of the study [47]. The second part of this study was a narrative review and pooled analysis for the current body of evidence on dental students’ knowledge, attitudes, and behaviours towards oral hygiene in the Arab region, which were assessed using the HU-DBI.

### 2.2. Setting

The study used a non-probability technique, convenience sampling, to recruit participants from the target population who were the dental students in the Lebanese Republic (Lebanon), the Syrian Arab Republic (Syria), and the Republic of Tunisia (Tunisia) between November 2019 and May 2020—the academic year 2019/2020.

In Lebanon, data were collected from three universities that had undergraduate dental degree programs, i.e., Beirut Arab University (BAU), Lebanese University (LU), and Saint Joseph University of Beirut (USJ). In Syria, data was collected from a single private university in Damascus, the Syrian Private University (SPU); while in Tunisia, data was collected from the only university that had a dental school, the University of Monastir (UM).

### 2.3. Participants

The target population of this study comprised students of dental degree programs in the three participating countries. The undergraduate students were included, while the postgraduate students and residents were excluded. The required sample size was calculated using Epi-Info^TM^ (CDC. Atlanta, GA, USA. 2019) and following the default assumptions of outcome probability 50%, confidence level 95%, and error margin 5% [48]. According to the target population size, between 305–340 dental students were required from each participating country [49,50].

The undergraduate dental degree programs in Lebanon and Syria last for five years (10 semesters), while the program in Tunisia lasts for six years (12 semesters). The preclinical subjects are extended over the first six semesters; therefore, the first, second, and third years were denoted as “preclinical”, and the fourth, fifth, and sixth years were denoted as “clinical” [51,52,53].

### 2.4. Instrument

A bi-lingual SAQ was used to collect data digitally from the participating students through KoBoToolbox (Harvard Humanitarian Initiative, Cambridge, MA, USA) [54]. The Arabic version of HU-DBI developed and validated by Daou et al. 2018 was used in addition to the English and French versions [55,56]. All original items of HU-DBI are binary questions, with “Agree” or “Disagree” answers, out of which 12 items are used to compute the overall HU-DBI score. One point was given for each “agree” response for items no. 4, 9, 11, 12, 16, and 19, and for each “disagree” responses for items no. 2, 6, 8, 10, 14, and 15. Therefore, the HU-DBI score ranges between 0 (worst score) to 12 (best score). The oral health knowledge score was dependent on items no. 2, 8, 10, 15, and 19, while oral health attitudes score was dependent on items no. 6, 11, and 14, and behaviours score on items no. 4, 9, 12, and 16 [57] (Table A1).

An overall score of 12 comprises the knowledge + attitudes + behaviours components, with a higher score indicating better oral hygiene [41,57]. Three items of risk behaviours were added to the original items of HU-DBI; (a) tobacco smoking (I smoke cigarettes once every week at least), (b) drinking alcohol (I drink alcohol once every week at least), and (c) internet addiction (I find myself using my smartphone or computer longer than I planned to).

### 2.5. Ethics

The study protocol was reviewed and approved by the Ethical Committee of Faculty of Medicine, Masaryk University on 20 November 2019 with reference number 48/2019. The inception and conduction of this study were guided by the declaration of Helsinki of research on human subjects, and the General Data Protection Regulation principles had guided the process of data storage and management [58,59].

Each participant was required to provide their informed consent digitally prior to their participation in the study. The participants were able to withdraw from the study at any time without the need to justify their decision or save any of their information or answers. Retrospective identification of the study participants was not possible because no personal identifying data was collected. Additionally, the participants were not offered any incentive to participate in this study. 

### 2.6. Analyses

The Statistical Package executed all statistical tests for the Social Sciences (SPSS) version 28.0 (SPSS Inc., Chicago, IL, USA, 2021) [60]. Descriptive statistics were carried out to describe the sociodemographic characteristics, risk behaviours, and HU-DBI responses of the participants using frequencies (*n*), percentages (%), mean and standard deviation (*µ* ± SD). Subsequently, inferential statistics were performed to evaluate the association between independent variables (sociodemographic characteristics and risk behaviours) and dependent variables (knowledge, attitudes, behaviours, and HU-DBI score) using the Chi-squared test (*χ*^2^), Mann–Whitney test (*U*) and Kruskal–Wallis test (*H*). The year-over-year (YOY) analysis was performed using a pairwise comparison (Mann–Whitney test) for the consecutive academic years to evaluate the gains in HU-DBI scores. A bivariate correlation analysis was performed to explore the association between HU-DBI score and Arab countries’ economic rank [61]. All inferential tests were carried out with confidence level (*CI*) 95% and significance level (*p*) < 0.05. 

## 3. Results

### 3.1. Sample Characteristics

A total of 1430 students provided their consent to participate and responded to the SAQ, out of which 316 (22.1%) were from Lebanon, 561 (39.2%) from Syria, and 553 (38.7%) from Tunisia. The overall female-to-male ratio was 39.2% vs. 60.8%, with the highest proportion of females in Tunisia (75.9%), while the highest proportion of males was in Syria (57.4%). The most represented academic year was the second year (21.5%), while the least represented was the sixth year (6.4%) which was solely present in Tunisia. Preclinical students represented 57.8% of the sample, with the highest proportion of preclinical students in Lebanon (66.1%), followed by Syria (59.4%) and Tunisia (51.5%) (Table 1).

Regarding the risk behaviours, internet addiction was the most prevalent behaviour (87%), followed by tobacco smoking (24.5%) and alcohol drinking (7.2%). Tunisia had the highest proportion of internet addiction (93.9%), and Syria had the highest proportion of tobacco smoking (32.1%). There was no significant difference between Syrian (7.7%) and Tunisian students (6.7%) in terms of alcohol drinking (*p* = 0.529). The item of alcohol drinking was not included in the Lebanese form due to cultural sensitivity concerns (Table 2).

### 3.2. HU-DBI Responses

Item No. 3 (concerns of discolouration) had the highest level of agreement (94.5%), followed by items No. 12 (post-brushing checking) and No. 13 (concerns of halitosis), 91.5% and 91.2%, respectively. On the other hand, item No. 8 (declining oral health) had the lowest level of agreement (25.9%), followed by item No. 10 (toothbrush education) and item No. 2 (gingival bleeding tendency), 31% and 32.1%, respectively. Twelve items were significantly different across the three countries, as Lebanon had the highest disagreement level with items No. 2 (79.4%) and No. 15 (63%) and the highest agreement level with items No. 9 (76.6%) and No. 16 (16.5%). Syria had the highest disagreement level with items No. 4 (63.8%) and No. 12 (11.8%), while the highest agreements level with items No. 18 (44.2%), No. 19 (54.7%), and No. 20 (66.8%). Tunisia had the highest agreement level with item No. 15 (59.5%) (Table 3).

A gradual ascending pattern was statistically significant (*p* < 0.001) in item No. 8 as the first year had the lowest level of disagreement (65.1%) and the sixth year had the highest level of agreement (85.9%). Similarly, item No. 11 (1st year: 13.9% vs. 6th Year: 46.7%) had a statistically significant ascending gradient (Table S1).

The male students had significantly higher agreement levels than their female peers for the items No. 1 (83.2% vs. 74.1%), No. 4 (41.4% vs. 30%), No. 17 (25% vs. 16.9%), No. 18 (39% vs. 31.4%), and No. 20 (63.8% vs. 58.5%), respectively. On the other hand, the female students had significantly higher agreement levels than their male peers for the items No. 5 (31% vs. 25.3%) and No. 12 (93.6% vs. 88.4%), respectively.

The clinical students had significantly higher agreement levels than their preclinical peers for the items No. 5 (33.3% vs. 25.4%), No. 9 (70.5% vs. 64.9%), No. 11 (27.2% vs. 15.5%), No. 16 (12.4% vs. 7.5%), and No. 20 (66% vs. 56.6%), respectively. On the contrary, the preclinical students had significantly higher agreement levels than their clinical peers for items No. 17 (24.8% vs. 13.6%) and No. 18 (40.9% vs. 25.5%), respectively. Moreover, the clinical students had a significantly higher disagreement level than their preclinical peers for the items No. 2 (74.3% vs. 63.2%), No. 8 (80.1% vs. 69.6%), No. 10 (77.3% vs. 63%), No. 14 (42.5% vs. 33.9%), No. 15 (53.9% vs. 48%), respectively (Table 4).

In Lebanon, no statistically significant differences were found between males or females in terms of their answers to the original HU-DBI items; however, the male students had significantly higher agreement levels than their female peers with item No. 4 in Syria (43.8% vs. 25.9%) and Tunisia (44.4% vs. 33.8%), respectively. Syrian males were significantly more agreeable than their female peers with items No. 1 (86.3% vs. 71.1%) and No. 7 (15.8% vs. 8.8%), while they were significantly less agreeable with item No. 5 (25.5% vs. 36.8%), respectively. Additionally, Tunisian males had significantly higher agreement levels than their female peers with items No. 11 (30.8% vs. 21.9%), No. 17 (32.3% vs. 14.8%), and No. 18 (36.1% vs. 26%), respectively. On the contrary, Tunisian females had a significantly higher disagreement level than their male peers with item No. 10 (77.9% vs. 67.7%), respectively (Tables S2–S4).

The clinical students had a significantly higher agreement level than their preclinical peers with item No. 2 in Lebanon (88.8% vs. 74.6%), Syria (75.4% vs. 61.3%), and Tunisia (67.5% vs. 57.2%), respectively. Similarly, the clinical students had a significantly higher disagreement level than their preclinical peers with item No. 10 in Lebanon (86.9% vs. 68.4%), Syria (64.5% vs. 56.2%), and Tunisia (84.3% vs. 67%), respectively (Tables S2–S4).

### 3.3. HU-DBI Scores

The overall HU-DBI score in the three participating countries was 6.31 ± 1.84, with Lebanon having the highest overall score (6.67 ± 1.83), followed by Syria (6.38 ± 1.83), and Tunisia (6.05 ± 1.83). The difference between males (6.41 ± 1.74) and females (6.25 ± 1.90) was not statistically significant in any of the participating countries. The first year had the lowest score (5.75 ± 1.95), while the fifth (6.83 ± 1.73) and sixth (6.91 ± 1.53) years had the highest score. The smokers (6.37 ± 1.85) and internet addicts (6.66 ± 1.74) had significantly (*p* = 0.016 and 0.007, respectively) higher scores than non-smokers (6.15 ± 1.80) and non-addicts (6.26 ± 1.85) (Table 5).

**Figure 1:**
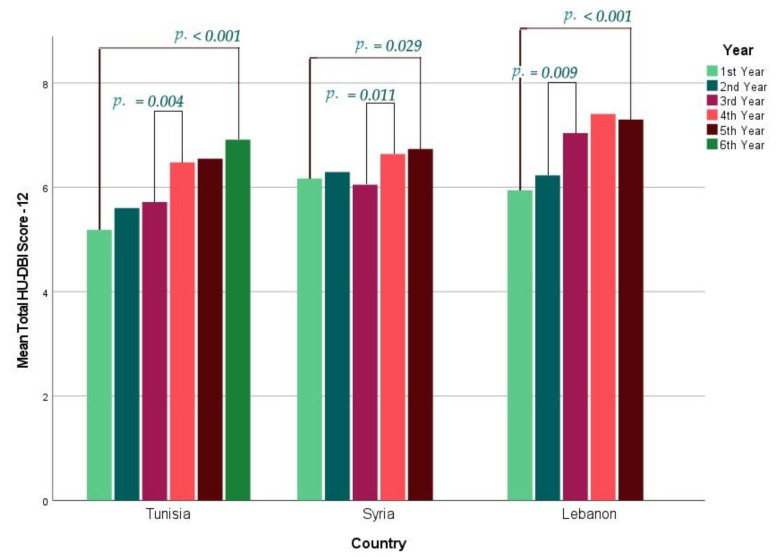
Mean HU-DBI Score of the Participating Dental Students Stratified by Academic Year, 2019/2020, (*n* = 1430).

In Lebanon, the first year had the lowest score (5.94 ± 1.92) and the fourth year had the highest score (7.41 ± 1.79). In Syria, the third year had the lowest score (6.17 ± 1.91) and the fifth year had the highest score (6.73 ± 1.73). In Tunisia, the first year had the lowest score (5.18 ± 1.89) and the sixth year had the highest score (6.91 ± 1.53). The difference between the first and last year was statistically significant (*p* < 0.001) in all participating countries (Figure 1).

On analysing the year-over-year (YOY) changes of HU-DBI score, the only significant (*p* = 0.009) improvement among Lebanese students was apparent between the second year (6.23 ± 1.46) and the third year (7.04 ± 1.72). In Syria, the only significant YOY change (*p* = 0.011) occurred between the third (6.17 ± 1.91) and the fourth year (6.64 ± 1.69). Similarly, the only significant YOY change (*p* = 0.004) among Tunisian students occurred between the third year (5.72 ± 1.84) and the fourth year (6.48 ± 1.70) (Table 6).

In Lebanon, the YOY analysis revealed that the sole significant (*p* = 0.001) improvement in knowledge occurred between the second and the third years, while the sole significant (*p* = 0.042) improvement in behaviours occurred following the first year. The sole significant (*p* = 0.003) improvement in knowledge was observed between the third and fourth years in Syria. In contrast, the sole significant (*p* = 0.022) improvement in attitudes occurred between the second and third years. In Tunisia, the significant improvements in knowledge occurred between the third and fourth years (*p* = 0.050) and following the first year (*p* = 0.006), while the sole significant (*p* = 0.002) improvement in attitudes occurred between the third and fourth years (Tables S5–S7).

**Figure 2:**
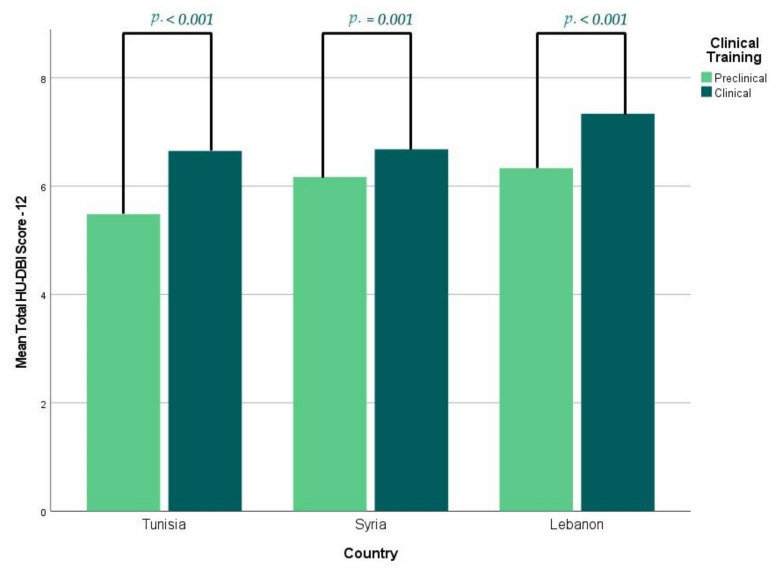
Mean HU-DBI Score of the Participating Dental Students Stratified by Clinical Training, 2019/2020, (*n* = 1430).

In general, the clinical students (6.78 ± 1.70) had a significantly (*p* < 0.001) higher overall score than their preclinical peers (5.97 ± 1.86), and this trend was significant (*p* < 0.001, = 0.001, and <0.001) in Lebanon (7.34 vs. 6.33), Syria (6.68 vs. 6.17), and Tunisia (6.65 vs. 5.48), respectively (Figure 2).

### 3.4. Determinants of Knowledge, Attitudes and Behaviours

The HU-DBI knowledge score was 3.06 ± 1.23 (0–5), with Lebanese students having the highest knowledge score (3.34 ± 1.16), followed by Syrians (3.07 ± 1.25) and Tunisians (2.89 ± 1.21). There was no significant difference in HU-DBI knowledge score across sex, smoking or drinking alcohol.

The HU-DBI attitudes score was 1.22 ± 0.84 (0–3), with Tunisian students having the highest attitudes score (1.26 ± 0.81), followed by Lebanese (1.21 ± 0.83) and Syrians (1.19 ± 0.86). There was no significant difference in HU-DBI attitudes score across sex, internet addiction or drinking alcohol.

The HU-DBI behaviours score was 2.03 ± 0.75 (0–4), with Lebanese students having the highest attitudes score (2.12 ± 0.73), followed by Syrians (2.11 ± 0.77) and Tunisians (1.89 ± 0.73). There was no significant difference in HU-DBI behaviours score across tobacco smoking, drinking alcohol or internet addiction. Male students (2.10 ± 0.77) had a significantly (*p* = 0.008) higher behaviour score than their female peers (1.98 ± 0.73) (Table 5).

The clinical students (3.33 ± 1.11) had a significantly (*p* < 0.001) higher knowledge score than the preclinical students (2.87 ± 1.27). This difference has been found in Lebanon (3.70 vs. 3.15), Syria (3.33 vs. 2.90), and Tunisia (3.18 vs. 2.82). Smokers, alcohol drinkers, and internet addicts were found to have a lower knowledge score than their counterparts in the three participating countries.

The clinical students (1.36 ± 0.84) had a significantly (*p* < 0.001) higher attitudes score than the preclinical students (1.12 ± 0.82). This difference was found in Lebanon (1.31 vs. 1.16), Syria (1.23 vs. 1.12), and Tunisia (1.30 vs. 1.09). Smokers have a lower attitudes score than their counterparts in the three participating countries.

The clinical students (2.09 ± 0.74) had a significantly (*p* = 0.005) higher behaviours score than the preclinical students (1.98 ± 0.76). This trend has been found in Lebanon (2.33 vs. 2.02), Syria (2.18 vs. 2.06), and Tunisia (1.93 vs. 1.86) (Tables S8–S10).

### 3.5. Determinants of Regular Dental Attendance

A total of 637 (44.5%) students reported being regular dental attendants as they visited the dentist for a check-up at least once a year. The rate was not significantly different between males (46.9%) vs. females (43%) or clinical (46.3%) vs. preclinical students (43.3%). Lebanese students had the highest attendance rate (67.1%), followed by Syrians (52%), and Tunisians (24.1%). Smokers (49.6%) and alcohol drinkers (50%) were significantly (*p* = 0.029 and 0.024) more likely to visit the dentist regularly than non-smokers (42.9%) and non-drinkers (37.2%). The overall HU-DBI score was significantly (*p* < 0.001) higher among regular attendants than their peers, 6.89 vs. 5.85, respectively. Similarly, the regular attendants had higher knowledge (3.47 vs. 2.74), attitudes (1.27 vs. 1.19), and behaviours (2.15 vs. 1.93) scores than their peers, respectively (Table 7).

On running multinomial logistic regression for the regular dental attendance, non-smokers, non-drinkers, and internet addicts had an adjusted odds ratio (AOR) of 0.667, 0.603, and 1.705 times for being regular dental attendants compared to their peers. The students with higher knowledge and behaviours scores were 1.525 and 1.367 times more likely to visit the dentist once a year (Table 8).

### 3.6. HU-DBI Scores in the Arab Region 2000–2020

On reviewing the published literature on oral health-related knowledge, attitudes, and behaviours of Arab dental students assessed by the HU-DBI, fourteen studies have been previously published with regard to eight countries, i.e., Egypt [57,62], Jordan [63,64], Kuwait [65], Palestine [66], Saudi Arabia [67,68,69], Sudan [70], United Arab Emirates (UAE) [71,72,73], and Yemen [19], in addition to the three countries included within the current report, i.e., Lebanon, Syria, and Tunisia (Table S11).

**Figure 3:**
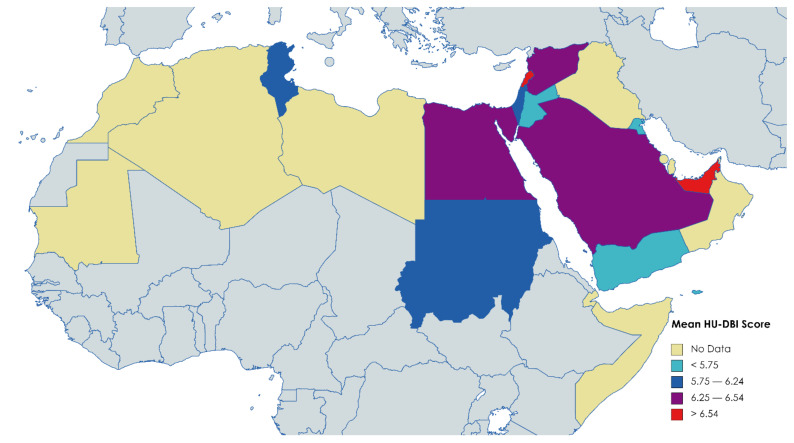
Distribution of HU-DBI Score levels among Dental Students in the Arab League Member States, 2004–2020, (*n* = 6941).

A total of 6941 dental students had been surveyed, with a mean overall score of 6.21. The lowest HU-DBI score was reported among Yemeni students (5.06) by Halboub et al. 2015, while the highest score was reported among Emirati students (9.45) by Kawas et al. 2009 [19,71] (Figure 3).

The first published study was from Jordan by Al-wahadni et al. 2004, and the mean score of the studies published between 2000–2010 was 5.82, while the mean score of the period 2011–2020 was 6.27 [63] (Table 9).

In Egypt, Al-wesabi et al. 2019 found a significant increase in students’ knowledge, attitudes, and behaviours while progressing from the first year to the final year [57]. Similarly, Abu Alregal et al. 2018 found that clinical students had significantly higher levels of oral health knowledge and attitudes than preclinical students; however, the level of behaviour was similar, indicating that knowledge and attitudes may not be able to predict oral health behaviours among dental students in Egypt [62].

Al-wahadni et al. 2004 revealed significant differences between dental surgery, dental hygiene, and dental technology students in Jordan, thus suggesting that there could be a role for dental curricula in shaping the students’ attitudes and behaviours [63]. In a later Jordanian study, Al-omiri et al. 2012 found that female and clinical students had better oral health attitudes and behaviours than male and preclinical students, respectively [64]. Similarly, dental students in Kuwait had better oral health attitudes and behaviours than other healthcare students (medicine, pharmacy, and allied health professions), and Kuwaiti female and clinical students had significantly higher levels of attitudes and behaviours than their counterparts [65]. Palestinian female students had significantly better oral health attitudes and behaviours than their male peers [66].

In Saudi Arabia, direct comparison between females and males was not possible since the Saudi higher education system is gender-segregated [75]. While clinical male students had better oral health attitudes and behaviours than preclinical male students, clinical and preclinical female students did not have different oral health attitudes or behaviours [67,69]. The largest study was conducted by Khalid et al., 2016 which included 1243 students from nine Sudanese universities and revealed the superiority of female and clinical students in terms of oral health knowledge and attitudes compared to their counterparts [70]. Similarly, female and clinical students in UAE had better oral health knowledge and attitudes [71,72,73]. Rahman et al., 2013 revealed that better oral health attitudes were significantly associated with lower plaque scores and moderate plaque and gingival bleeding scores, thus emphasising the need for more preventive measures in dental curricula [73]. In Yemen, female and public university students had better oral health outcomes than male students and private universities students, respectively, even though the differences across education levels were insignificant [19].

A weak positive correlation was found on performing a correlation test between the mean HU-DBI score of Arab dental students and their countries economic rank according to the World Bank (Spearman’s ρ = 0.296; *p* < 0.001) (Table 10).

## 4. Discussion

In the present study, the mean HU-DBI score of dental students in the participating countries was 6.31 ± 1.84, with Lebanon having the highest score (6.67 ± 1.83), followed by Syria (6.38 ± 1.83), and Tunisia (6.05 ± 1.83). Male students (6.41 ± 1.74) and clinical students (6.78 ± 1.70) had higher HU-DBI scores than female students (6.25 ± 1.90) and preclinical students (5.97 ± 1.86), respectively. Tobacco smoking, alcohol drinking, and problematic internet use were associated with lower HU-DBI scores.

A prospective cohort study assessed oral health outcomes of French dental students and found that their frequency and duration of toothbrushing had increased significantly during their study at Paris VII University [76]. The use of adjuvants such as toothpicks, water-picks, and silk threads, the use of toothbrushes for only six months or below, and regular check-up visits increased significantly from the first to the second recording, which were four years apart [76]. Moreover, the clinical parameters such as the simplified oral hygiene index (OHI-S) of Greene & Vermillion, the gingival index (GI) of Löe and Silness, decreased significantly throughout the study years, indicating empirical improvement of oral hygiene [76,77,78]. However, the decayed, missed, filled teeth (DMFT) score had increased significantly from the first to the second recording; the number of filled teeth was the main reason for this increase (62.57%), thus suggesting better utilisation of conservative services [76]. In another cohort study, Peretz et al. 2002 found that dental students’ dental anxiety levels had decreased significantly during their undergraduate education years, especially among females, which could be attributed to their dental curricula and the clinical experience they gained during their studies [79]. Therefore, the impact of dental curricula can be echoed by dental students’ oral health knowledge and attitudes, which reflect how much they appreciate prevention and practice it in their daily lives [80]. Given this notion, it should be hypothesised that the dental students of advanced years–clinical students—would have better oral health knowledge, attitudes, and behaviours compared with the students of early years–preclinical students.

In our study, the superiority of clinical students was observed in the three participating countries and all oral health domains: knowledge, attitudes, and behaviours. Our findings are consistent with what was concluded by studies that were carried out in other Arab countries, e.g., Egypt [57,62], Jordan [64], Kuwait [65], Saudi Arabia [67,69], Sudan [70], and United Arab Emirates [71,73] and even non-Arab countries, e.g., Croatia [45], Greece [81], Lithuania [46], Turkey [18,80], Nigeria [82], Japan [22], South Korea [83], Pakistan [84], and Peru [17] on dental students using HU-DBI. On the other hand, preclinical students had better oral health attitudes and behaviours than their clinical peers as assessed by HU-DBI in Germany [85] and India [20,86].

For a better understanding of the role of the dental curriculum in improving oral health knowledge and attitudes of dental students, we performed a year-over-year (YOY) analysis to track the gradual changes in oral health knowledge, attitudes and behaviours throughout dental education years. The YOY analysis revealed that the sole significant increase in the HU-DBI score occurred between the second and the third year in Lebanon, and between the third and the fourth year in Syria and Tunisia. In Lebanon, the course of “Preventive and Public Health Dentistry” is delivered in the second academic year [51]. According to the Syrian Private University (SPU) study plan, the course “Dental Public Health and Preventive Dentistry” is delivered in the third year [52]. Similarly, in Tunisia, the course “Oral Hygiene and Prevention” is delivered during the third year [53]. Therefore, this significant increase in oral health knowledge and attitudes that occurred following the course of dental public health suggests that this course was the main source of theoretical knowledge and practical skills relating to oral hygiene.

Female students represented the majority of participants in the present study, which might reflect the actual gender distribution of dental students in Arab countries; however, there is a lack of information about dental students’ demographic characteristics in the region. The HU-DBI differences between females and males were not statistically significant among our participants; nevertheless, there was a trend favouring males, especially in terms of oral health behaviours. In Lebanon, the differences across genders were entirely absent, while few differences were statistically significant in Syria and Tunisia. Our findings are in agreement with previous studies that found that male students had significantly better oral health than females, e.g., Croatia [45], Lithuania [46], and India [20]. In contrast to our results, several studies using HU-DBI in Arab countries, e.g., Jordan [64], Kuwait [65], Palestine [66], Sudan [70], United Arab Emirates [71,72,73], and Yemen [19] and non-Arab countries, e.g., Greece [81], India [86,87], and Turkey [88] found that female dental students had better oral health than their male counterparts.

Regarding the general health-related behaviours, Syria had the highest prevalence of tobacco smoking, while Tunisia had the highest prevalence of problematic internet use. The differences between the smoker and non-smoker students in Lebanon and Syria were not statistically significant; however, the HU-DBI score of smokers in Tunisia was significantly (*p* = 0.001) lower than non-smokers, 5.56 vs. 6.16, respectively. Several studies for adolescents and adults in Finland, Japan, and Iran revealed a significant correlation between smoking and poor oral hygiene habits, thus suggesting that anti-smoking activities should be incorporated in comprehensive oral health promotion [89,90,91,92]. Additionally, tobacco smoking is a predictor for periodontal disease and negative oral health outcomes; for instance, Setia et al, 2014 found that tobacco smoking was significantly correlated with self-perceived halitosis among undergraduate dental students in India [92,93]. In Japan, Haresaku et al. 2010 evaluated the impact of the smoking curriculum with the no-smoking policy recently introduced to the undergraduate dental curriculum. They found that smoking rates decreased significantly from 35% to 26% among students after three years of introducing these changes [94]. Another study from Belgium recommended that dental curricula emphasise the effectiveness of anti-smoking activities in theoretical lectures and practical lessons because knowledge of smoking harms is not sufficient for improving dental students’ attitudes [95]. The societal role of dental students as future healthcare workers needs to be insisted in dental curricula. Dentists are efficient in improving oral hygiene behaviours of their patients and their general health beliefs, attitudes, and behaviours, e.g., healthy nutrition, physical activity, smoking cessation, moderate drinking, and preventive medicine and vaccination [29,90,91,96,97,98,99,100,101].

Internet addiction (problematic internet use) refers to a range of repetitive activities, e.g., excessive video gaming, online shopping, social media use, and cybersex that limits the ability to control the amount of time spent online [102]. However, the prevalence of internet addiction is rising worldwide. We still lack standardised methods for its population-level surveillance that are vital for evidence-informed interventions targeting this growing pandemic [103]. The impact of internet addiction on health behaviours such as nutrition, physical activity, and sleep quality has been widely studied among several adolescent and adult groups; however, there is a lack of evidence on the relationship between internet addiction and health behaviours among healthcare students and healthcare workers including dental students and dentists [31,103]. The present study is the first to shed light on the potential correlation between internet addiction and oral health attitudes and behaviours, thus calling for further investigation to better understand the interactions between oral hygiene and internet addiction.

On reviewing the current literature, we found a weak correlation between the mean HU-DBI score of Arab dental students and the economic rank of their countries, thus suggesting that socioeconomic index can be a functional predictor for oral health outcomes in Arab countries that should be considered in the future research. The country’s economic capacity where the dental students live/study was found to be a robust ecological predictor for dental students’ attitudes towards health behaviours [100].

### 4.1. Strengths

To the best of the authors’ knowledge, this study is the first to provide evidence on the oral health of dental students in Lebanon, Syria, and Tunisia. The present study also provided a literature review for the oral health of Arab dental students for the first time. The participating students’ identity was anonymous to control Hawthorne’s effect. The participants did not receive incentives that may have caused information bias. The Arabic version of HU-DBI used in this study had been thoroughly tested and exhibited excellent psychometric properties. 

### 4.2. Limitations

The first limitation of this study is its cross-sectional design that hindered the longitudinal follow-up of the participating students to track changes in their oral health knowledge and attitudes. The second limitation is the lack of clinical examinations that could have revealed interactions between oral health knowledge, attitudes and behaviours and actual clinical outcomes. The third limitation is the unbalanced distribution between females/males and preclinical/clinical students, which can be attributed to the recruitment strategy that was based on convince sampling.

### 4.3. Implications

The findings of this study imply that future studies on the oral health of dental students should consider a prospective follow-up, which means that they should be designed as cohort rather than cross-sectional studies to validate the hypotheses related to curriculum impact. The dental public health and preventive dentistry courses need to be integrated in earlier years as they can help raise students’ awareness and improve their attitudes and behaviours. The common risk factor approach should be implemented in dental curricula of Arab universities as the future dentists in the region can improve the health-related behaviours of their patients, e.g., nutrition, smoking, and physical activity through counselling.

## 5. Conclusions

In conclusion, oral health-related knowledge, attitudes and behaviours of dental students in the three participating Arabic countries were satisfactory. The mean HU-DBI score was 6.31 ± 1.84, with Lebanon having the highest score (6.67 ± 1.83), followed by Syria (6.38 ± 1.83) and Tunisia (6.05 ± 1.83). Clinical students (6.78 ± 1.70) had higher HU-DBI scores than their preclinical peers (5.97 ± 1.86). The year-over-year analysis revealed that dental public health and preventive dentistry courses had significantly and positively impacted the undergraduate students’ knowledge, attitudes, and behaviours. The gender-based differences were not statistically significant, with a modest trend favouring males, especially in terms of oral health behaviours. Tobacco smoking, alcohol drinking, and problematic internet use were associated with lower HU-DBI scores. In the Arab world, the economic rank of the country where the dental students live/study was weakly correlated with the students’ mean HU-DBI score.

## Figures and Tables

**Table 1:** Sociodemographic Characteristics of the Participating Dental Students from Lebanon, Syria, and Tunisia, 2019/2020, (*n* = 1430).

Variable	Group	Lebanon (*n* = 316)	Syria (*n* = 561)	Tunisia (*n* = 553)	Total (*n* = 1430)	*p*
**Sex**	Female	210 (66.5%)	239 (42.6%)	420 (75.9%)	869 (60.8%)	**<0.001**
	Male	106 (33.5%)	322 (57.4%)	133 (24.1%)	561 (39.2%)	**<0.001**
**Academic ** **Year**	1st Year	71 (22.5%)	107 (19.1%)	103 (18.6%)	281 (19.7%)	0.354
	2nd Year	87 (27.5%)	109 (19.4%)	111 (20.1%)	307 (21.5%)	**0.012**
	3rd Year	51 (16.1%)	117 (20.9%)	71 (12.8%)	239 (16.7%)	**0.002**
	4th Year	37 (11.7%)	130 (23.2%)	94 (17%)	261 (18.3%)	**<0.001**
	5th Year	70 (22.2%)	98 (17.5%)	82 (14.8%)	250 (17.5%)	**0.024**
	6th Year	N/A	N/A	92 (16.6%)	92 (6.4%)	N/A
**Clinical ** **Training**	Preclinical	209 (66.1%)	333 (59.4%)	285 (51.5%)	827 (57.8%)	**<0.001**
	Clinical	107 (33.9%)	228 (40.6%)	268 (48.5%)	603 (42.2%)	**<0.001**

Chi-squared (*χ^2^*) test was used with a significance level *p* ≤ 0.05. The significant values are in bold font.

**Table 2:** Risk Behaviours of the Participating Dental Students from Lebanon, Syria, and Tunisia, 2019/2020, (*n* = 1430).

Variable	Group	Lebanon (*n* = 316)	Syria (*n* = 561)	Tunisia (*n* = 553)	Total (*n* = 1430)	*p*
**Tobacco Smoking**	Yes	69 (21.8%)	180 (32.1%)	102 (18.4%)	351 (24.5%)	**<0.001**
	No	247 (78.2%)	381 (67.9%)	451 (81.6%)	1079 (75.5%)	**<0.001**
**Alcohol Drinking**	Yes	N/A	43 (7.7%)	37 (6.7%)	80 (7.2%)	0.529
	No	N/A	518 (92.3%)	516 (93.3%)	1034 (92.8%)	0.529
**Internet Addiction**	Yes	280 (88.6%)	445 (79.3%)	519 (93.9%)	1244 (87%)	**<0.001**
	No	36 (11.4%)	116 (20.7%)	34 (6.1%)	186 (13%)	**<0.001**

Chi-squared (*χ^2^*) test was used with a significance level *p* ≤ 0.05. The significant values are in bold font.

**Table 3:** Responses of the Participating Students to the Individual HU-DBI Items Stratified by Country, 2019/2020, (*n* = 1430).

Item	Response	Lebanon (*n* = 316)	Syria (*n* = 561)	Tunisia (*n* = 553)	Total (*n* = 1430)	*p*
Item No. 1	Agree	248 (78.5%)	448 (79.9%)	415 (75%)	1111 (77.7%)	0.145
Item No. 2	Disagree	251 (79.4%)	376 (67%)	344 (62.2%)	971 (67.9%)	**<0.001**
Item No. 3	Agree	297 (94%)	527 (93.9%)	528 (95.5%)	1352 (94.5%)	0.466
Item No. 4	Agree	89 (28.2%)	203 (36.2%)	201 (36.3%)	493 (34.5%)	**0.028**
Item No. 5	Agree	60 (19%)	170 (30.3%)	181 (32.7%)	411 (28.7%)	**<0.001**
Item No. 6	Disagree	206 (65.2%)	348 (62%)	368 (66.5%)	922 (64.5%)	0.277
Item No. 7	Agree	16 (5.1%)	72 (12.8%)	70 (12.7%)	158 (11%)	**0.001**
Item No. 8	Disagree	247 (78.2%)	409 (72.9%)	403 (72.9%)	1059 (74.1%)	0.168
Item No. 9	Agree	242 (76.6%)	420 (74.9%)	300 (54.2%)	962 (67.3%)	**<0.001**
Item No. 10	Disagree	236 (74.7%)	334 (59.5%)	417 (75.4%)	987 (69%)	**<0.001**
Item No. 11	Agree	53 (16.8%)	106 (18.9%)	133 (24.1%)	292 (20.4%)	**0.019**
Item No. 12	Agree	288 (91.1%)	495 (88.2%)	526 (95.1%)	1309 (91.5%)	**<0.001**
Item No. 13	Agree	291 (92.1%)	501 (89.3%)	512 (92.6%)	1304 (91.2%)	0.126
Item No. 14	Disagree	123 (38.9%)	215 (38.3%)	198 (35.8%)	536 (37.5%)	0.573
Item No. 15	Disagree	199 (63%)	299 (53.3%)	224 (40.5%)	722 (50.5%)	**<0.001**
Item No. 16	Agree	52 (16.5%)	65 (11.6%)	20 (3.6%)	137 (9.6%)	**<0.001**
Item No. 17	Agree	61 (19.3%)	121 (21.6%)	105 (19%)	287 (20.1%)	0.521
Item No. 18	Agree	87 (27.5%)	248 (44.2%)	157 (28.4%)	492 (34.4%)	**<0.001**
Item No. 19	Agree	122 (38.6%)	307 (54.7%)	210 (38%)	639 (44.7%)	**<0.001**
Item No. 20	Agree	201 (63.6%)	375 (66.8%)	290 (52.4%)	866 (60.6%)	**<0.001**

Chi-squared (*χ*^2^) test was used with a significance level *p* ≤ 0.05. The significant values are in bold font.

**Table 4:** Responses of the Participating Students to the Individual HU-DBI Items Stratified by Gender and Clinical Training, 2019/2020, (*n* = 1430).

Item	Response	Female (*n* = 869)	Male (*n* = 561)	*p*	Preclinical (*n* = 827)	Clinical (*n* = 603)	*p*
Item No. 1	Agree	644 (74.1%)	467 (83.2%)	**<0.001**	654 (79.1%)	457 (75.8%)	0.140
Item No. 2	Disagree	587 (67.5%)	384 (68.4%)	0.722	523 (63.2%)	448 (74.3%)	**<0.001**
Item No. 3	Agree	830 (95.5%)	522 (93%)	**0.045**	778 (94.1%)	574 (95.2%)	0.359
Item No. 4	Agree	261 (30%)	232 (41.4%)	**<0.001**	283 (34.2%)	210 (34.8%)	0.812
Item No. 5	Agree	269 (31%)	142 (25.3%)	**0.021**	210 (25.4%)	201 (33.3%)	**0.001**
Item No. 6	Disagree	573 (65.9%)	349 (62.2%)	0.150	521 (63%)	401 (66.5%)	0.172
Item No. 7	Agree	79 (9.1%)	79 (14.1%)	**0.003**	89 (10.8%)	69 (11.4%)	0.685
Item No. 8	Disagree	639 (73.5%)	420 (74.9%)	0.574	576 (69.6%)	483 (80.1%)	**<0.001**
Item No. 9	Agree	572 (65.8%)	390 (69.5%)	0.146	537 (64.9%)	425 (70.5%)	**0.027**
Item No. 10	Disagree	618 (71.1%)	369 (65.8%)	**0.033**	521 (63%)	466 (77.3%)	**<0.001**
Item No. 11	Agree	166 (19.1%)	126 (22.5%)	0.124	128 (15.5%)	164 (27.2%)	**<0.001**
Item No. 12	Agree	813 (93.6%)	496 (88.4%)	**0.001**	757 (91.5%)	552 (91.5%)	0.996
Item No. 13	Agree	790 (90.9%)	514 (91.6%)	0.642	757 (91.5%)	547 (90.7%)	0.588
Item No. 14	Disagree	316 (36.4%)	220 (39.2%)	0.277	280 (33.9%)	256 (42.5%)	**0.001**
Item No. 15	Disagree	424 (48.8%)	298 (53.1%)	0.110	397 (48%)	325 (53.9%)	**0.028**
Item No. 16	Agree	78 (9%)	59 (10.5%)	0.334	62 (7.5%)	75 (12.4%)	**0.002**
Item No. 17	Agree	147 (16.9%)	140 (25%)	**<0.001**	205 (24.8%)	82 (13.6%)	**<0.001**
Item No. 18	Agree	273 (31.4%)	219 (39%)	**0.003**	338 (40.9%)	154 (25.5%)	**<0.001**
Item No. 19	Agree	387 (44.5%)	252 (44.9%)	0.886	354 (42.8%)	285 (47.3%)	0.094
Item No. 20	Agree	508 (58.5%)	358 (63.8%)	**0.043**	468 (56.6%)	398 (66%)	**<0.001**

Chi-squared (*χ*^2^) test was used with a significance level *p* ≤ 0.05. The significant values are in bold font.

**Table 5:** Knowledge, Attitudes, Behaviours and Total HU-DBI Score of the Participating Dental Students, 2019/2020, (*n* = 1430).

Variable	Group	Knowledge (0–5)	*p*	Attitudes (0–3)	*p*	Behaviours (0–4)	*p*	HU-DBI (0–12)	*p*
Sex	Female	3.06 ± 1.23	0.769	1.21 ± 0.82	0.734	1.98 ± 0.73	**0.008**	6.25 ± 1.90	0.162
	Male	3.07 ± 1.21		1.24 ± 0.87		2.10 ± 0.77		6.41 ± 1.74	
Academic Year	1st Year	2.71 ± 1.37	**<0.001**	1.14 ± 0.81	**<0.001**	1.90 ± 0.81	**0.002**	5.75 ± 1.95	**<0.001**
	2nd Year	2.92 ± 1.18		1.13 ± 0.82		1.98 ± 0.73		6.03 ± 1.78	
	3rd Year	2.99 ± 1.23		1.09 ± 0.85		2.08 ± 0.72		6.16 ± 1.85	
	4th Year	3.30 ± 1.15		1.29 ± 0.80		2.10 ± 0.75		6.69 ± 1.73	
	5th Year	3.37 ± 1.14		1.33 ± 0.87		2.13 ± 0.74		6.83 ± 1.73	
	6th Year	3.29 ± 0.94		1.65 ± 0.83		1.97 ± 0.72		6.91 ± 1.53	
Clinical Training	Preclinical	2.87 ± 1.27	**<0.001**	1.12 ± 0.82	**<0.001**	1.98 ± 0.76	**0.005**	5.97 ± 1.86	**<0.001**
	Clinical	3.33 ± 1.11		1.36 ± 0.84		2.09 ± 0.74		6.78 ± 1.70	
Country	Lebanon	3.34 ± 1.16	**<0.001**	1.21 ± 0.83	0.262	2.12 ± 0.73	**<0.001**	6.67 ± 1.83	**<0.001**
	Syria	3.07 ± 1.25		1.19 ± 0.86		2.11 ± 0.77		6.38 ± 1.83	
	Tunisia	2.89 ± 1.21		1.26 ± 0.81		1.89 ± 0.73		6.05 ± 1.83	
Tobacco Smoking	Yes	2.96 ± 1.25	0.068	1.12 ± 0.83	**0.006**	2.07 ± 0.78	0.457	6.15 ± 1.80	**0.016**
	No	3.10 ± 1.22		1.26 ± 0.84		2.01 ± 0.74		6.37 ± 1.85	
Alcohol Drinking	Yes	2.75 ± 1.39	0.109	1.11 ± 0.80	0.249	1.99 ± 0.77	0.816	5.85 ± 1.83	0.108
	No	3.00 ± 1.22		1.24 ± 0.85		2.00 ± 0.76		6.24 ± 1.83	
Internet Addiction	Yes	3.02 ± 1.23	**0.001**	1.23 ± 0.83	0.831	2.11 ± 0.70	0.175	6.26 ± 1.85	**0.007**
	No	3.34 ± 1.15		1.22 ± 0.88		2.02 ± 0.76		6.66 ± 1.74	

Mann–Whitney (*U*) and Kruskal–Wallis (*H*) tests were used with a significance level *p* ≤ 0.05. The significant values are in bold font.

**Table 6:** Pairwise Comparison of HU-DBI Total Score across Consecutive Academic Levels, 2019/2020, (*n* = 1430).

Pair	Lebanon (*n* = 316)		Syria (*n* = 561)		Tunisia (*n* = 553)	
	Mean Rank	*p*	Mean Rank	*p*	Mean Rank	*p*
1st Year vs. 2nd Year	76.80/81.71	0.495	107.01/109.96	0.726	99.40/115.02	0.062
2nd Year vs. 3rd Year	62.23/81.90	**0.004**	116.00/111.18	0.574	90.40/93.22	0.721
3rd Year vs. 4th Year 4th Year vs. 5th Year	42.89/46.72	0.482	111.99/134.81	**0.011**	71.92/91.37	**0.009**
	53.82/54.09	0.966	113.62/115.66	0.814	86.55/90.74	0.579
5th year vs. 6th year	*N/A*		*N/A*		82.59/91.88	0.214

Mann–Whitney (*U*) test was used with a significance level *p* ≤ 0.05. The significant values are in bold font.

**Table 7:** Predictors of Regular Dental Visits of the Participating Dental Students from Lebanon, Syria, and Tunisia, 2019/2020, (*n* = 1430).

Variable	Group	I Go to the Dentist for Regular Check-Up at Least Once a Year.		*p*
		No (*n* = 793)	Yes (*n* = 637)	
Sex	Female	495 (57%)	374 (43%)	0.153
	Male	298 (53.1%)	263 (46.9%)	
Clinical Training	Preclinical	469 (56.7%)	358 (43.3%)	0.263
	Clinical	324 (53.7%)	279 (46.3%)	
Country	Lebanon	104 (32.9%)	212 (67.1%)	**<0.001**
	Syria	269 (40%)	292 (52%)	
	Tunisia	420 (75.9%)	133 (24.1%)	
Tobacco Smoking	Yes	177 (50.4%)	174 (49.6%)	**0.029**
	No	616 (57.1%)	463 (42.9%)	
Alcohol Drinking	Yes	40 (50%)	40 (50%)	**0.024**
	No	649 (62.8%)	385 (37.2%)	
Internet Addiction	Yes	711 (57.2%)	533 (42.8%)	**0.001**
	No	82 (44.1%)	104 (55.9%)	
HU-DBI	Knowledge (0–5)	2.74 ± 1.21	3.47 ± 1.12	**<0.001**
	Attitudes (0–3)	1.19 ± 0.84	1.27 ± 0.84	0.108
	Behaviours (0–4)	1.93 ± 0.75	2.15 ± 0.74	**<0.001**
	Total (0–12)	5.85 ± 1.79	6.89 ± 1.74	**<0.001**

Chi-squared (*χ*^2^) and Mann–Whitney (*U*) tests were used with a significance level *p* ≤ 0.05. The significant values are in bold font.

**Table 8:** Multinomial Logistic Regression of Regular Dental Visits among the Participating Dental Students, 2019/2020, (*n* = 1430).

Predictor	B (SE)	Wald	AOR	CI 95%	*p*
Tobacco Smoking (No vs. Yes)	−0.404 (0.154)	6.93	0.667	0.494–0.902	0.008
Alcohol Drinking (No vs. Yes)	−0.506 (0.257)	3.89	0.603	0.364–0.997	0.049
Internet Addiction (No vs. Yes)	0.535 (0.184)	8.43	1.705	1.189–2.443	0.004
Knowledge	0.422 (0.056)	56.10	1.525	1.365–1.702	<0.001
Behaviours	0.313 (0.088)	12.58	1.367	1.150–1.625	<0.001

**Table 9:** Mean HU-DBI Score of Dental Students in the Arab League Member States, 2004 – 2020, (*n* = 6941).

Country	Author, Year of Study	Sample Size	University (City)	HU-DBI Score
Egypt	Al-wesabi et al., 2019 [57]	783	Private University (Cairo)	6.77
	Abu Alregal et al., 2018 [62]	896	Cairo University (Cairo)	6.33
Jordan	Al-wahadni et al., 2004 [63]	105	Jordan University of Science and Technology (Irbid)	6.38
	Al-omiri et al., 2012 [64]	580	University of Jordan (Amman)	5.20
Kuwait	Ali, 2016 [65]	141	Kuwait University (Kuwait)	5.74
Lebanon	Riad et al.	316	Multiple Universities (Beirut)	6.67
Palestine	Kateeb, 2006 [66]	260	Al-Quds University (Jerusalem)	6.13
Saudi Arabia	Baseer et al., 2011 [67]	351	Riyadh Colleges of Dentistry and Pharmacy (Riyadh)	6.54
	Kumar et al., 2011 [74]	26	Jazan University (Jazan)	6.65
	Moheet et al., 2013 [69]	112	University of Dammam (Dammam)	6.45
Sudan	Khalid et al., 2016 [70]	1243	Multiple Universities (Khartoum, Omdurman, Wad Madani)	6.24
Syria	Riad et al	561	Syrian Private University (Damascus)	6.38
Tunisia	Riad et al.	553	University of Monastir (Monastir)	6.05
UAE	Kawas et al., 2009 [71]	63	University of Sharjah (Sharjah)	9.45
	Hashim et al., 2012 [72]	279	Ajman University of Science and Technology (Ajman)	6.59
	Rahman et al., 2013 [73]	93	University of Sharjah (Sharjah)	7.32
Yemen	Halboub et al., 2015 [19]	579	Multiple Universities (Sana’a)	5.06

Lebanon: Beirut Arab University, Saint Joseph University of Beirut, and Lebanese University. Sudan: University of Khartoum, University of Gezira, National Ribat University, Africa International University, University of Science and Technology, University of Medical Sciences and Technology, Elrazi University, Al Neelain University, and National University—Sudan. Yemen: Sana’a University, and University of Science and Technology.

**Table 10:** Nonparametric Correlation of HU-DBI Score and Country Economic Rank, 2004–2020, (*n* = 6941).

		HU-DBI Score	Economic Rank
**HU-DBI Score**	Correlation Coefficient	1.000	0.296
	*p* (2-tailed)	N/A	<0.001
**Economic Rank**	Correlation Coefficient	0.296	1.000
	*p* (2-tailed)	<0.001	*N/A*

## Data Availability

The data that support the findings of this study are available from the corresponding author upon reasonable request.

## Supplementary Materials

The following supporting information can be downloaded at: https://www.mdpi.com/article/10.3390/ijerph19031658/s1, Table S1. Responses of the Participating Students to the Individual HU-DBI Items Stratified by Academic Year, 2019/2020, (*n* = 1430), Table S2. Lebanese Studentsʹ Responses to the Individual HU-DBI Items Stratified by Gender and Clinical Training, 2019/2020, (*n* = 316), Table S3. Syrian Studentsʹ Responses to the Individual HU-DBI Items Stratified by Gender and Clinical Training, 2019/2020, (*n* = 561), Table S4. Tunisian Studentsʹ Responses to the Individual HU-DBI Items Stratified by Gender and Clinical Training, 2019/2020, (*n* = 553), Table S5. Pairwise Comprison of Consecutive Academic Levels, Lebanon, 2019/2020, (*n* = 316), Table S6. Pairwise Comprison of Consecutive Academic Levels, Syria, 2019/2020, (*n* = 561), Table S7. Pairwise Comprison of Consecutive Academic Levels, Tunisia, 2019/2020, (*n* = 553), Table S8. Knowledge, Attitudes, Behaviours and Total HU-DBI Score of the Lebanese Dental Students, 2019/2020, (*n* = 316), Table S9. Knowledge, Attitudes, Behaviours and Total HU-DBI Score of the Syrian Dental Students, 2019/2020, (*n* = 561), Table S10. Knowledge, Attitudes, Behaviours and Total HU-DBI Score of the Tunisian Dental Students, 2019/2020, (*n* = 553), Table S11. HU-DBI Score of Dental Students in the Arab League Member States, 2006 – 2020, (*n* = 6941),

## Appendix A

**Table A1:** Modified version of the Hiroshima University—Dental Behavioural Inventory (HU-DBI).

No.	Question	Agree	Disagree
1	I do not worry much about visiting the dentist.	□	□
**2**	**My gum tends to bleed when I brush my teeth.**	**□**	**□**
3	I worry about the color of my teeth.	□	□
**4**	**I have noticed some white sticky deposits on my teeth.**	**□**	**□**
5	I use a child sized toothbrush.	□	□
**6**	**I think that I cannot help having false teeth when I am old.**	**□**	**□**
7	I am bothered by the color of my gum.	□	□
**8**	**I think my teeth are getting worse despite my daily brushing.**	**□**	**□**
**9**	**I brush each of my teeth carefully.**	**□**	**□**
**10**	**I have never been taught professionally how to brush.**	**□**	**□**
**11**	**I think I can clean my teeth well without using toothpaste.**	**□**	**□**
**12**	**I often check my teeth in a mirror after brushing.**	**□**	**□**
13	I worry about having bad breath.	□	□
**14**	**It is impossible to prevent gum disease with tooth brushing alone.**	**□**	**□**
**15**	**I put off going to dentist until I have a toothache.**	**□**	**□**
**16**	**I have used a dye to see how clean my teeth are.**	**□**	**□**
17	I use a toothbrush which has hard bristles.	□	□
18	I do not feel I have brushed well unless I brush with hard strokes.	□	□
**19**	**I feel I sometimes take too much time to brush my teeth.**	**□**	**□**
20	I have had my dentist tell me that I brush very well.	□	□
21	I find myself using my smartphone/compute longer than I planned.	□	□
22	I consume tobacco at least once a week.	□	□
23	I drink alcohol at least once a week.	□	□
24	I go to the dentist/hygienist for regular check-up at least once a year.	□	□

The questions No. 1–20 are the original HU-DBI items, and the questions in bold font are used to compute the overall score.
