# Supplementary material for: Oral Health-Related Knowledge, Attitudes and Behaviours of Arab Dental Students: Multi-National Cross-Sectional Study and Literature Analysis 2000–2020
Source: Int J Environ Res Public Health. 2022 Jan 31;19(3):1658. doi: 10.3390/ijerph19031658 (PMC8835654; doi:10.3390/ijerph19031658)
Supplement: Supplementary file 1 [file ijerph-19-01658-s001.zip › ijerph-1564832-supplementary.pdf]

Table S1. Responses of the Participating Students to the Individual HU-DBI Items Stratified by Academic Year, 2019/2020, (*n* = 1430)

Variable	Outcome	1st Year (*n* = 281)	2nd Year (*n* = 307)	3rd Year (*n* = 239)	4th Year (*n* = 261)	5th Year (*n* = 250)	6th Year (*n* = 92)	*Sig.*
**Item No. 1**	Agree	216 (76.9%)	242 (78.8%)	196 (82%)	208 (79.7%)	192 (76.8%)	57 (62%)	**0.005**
**Item No. 2**	Disagree	174 (61.9%)	197 (64.2%)	152 (63.6%)	190 (72.8%)	191 (76.4%)	67 (72.8%)	**0.001**
**Item No. 3**	Agree	264 (94%)	287 (93.5%)	227 (95%)	255 (97.7%)	231 (92.4%)	88 (95.7%)	0.134
**Item No. 4**	Agree	92 (32.7%)	101 (32.9%)	90 (37.7%)	94 (36%)	77 (30.8%)	39 (42.4%)	0.296
**Item No. 5**	Agree	71 (25.3%)	85 (27.7%)	54 (22.6%)	88 (33.7%)	78 (31.2%)	35 (38%)	**0.016**
**Item No. 6**	Disagree	184 (65.5%)	205 (66.4%)	133 (55.6%)	173 (66.3%)	162 (64.8%)	66 (71.7%)	**0.046**
**Item No. 7**	Agree	42 (14.9%)	25 (8.1%)	22 (9.2%)	28 (10.7%)	26 (10.4%)	15 (16.3%)	0.062
**Item No. 8**	Disagree	183 (65.1%)	215 (70%)	178 (74.5%)	201 (77%)	203 (81.2%)	79 (85.9%)	**< 0.001**
**Item No. 9**	Agree	170 (60.5%)	200 (65.1%)	167 (69.9%)	181 (69.3%)	193 (77.2%)	51 (55.4%)	**< 0.001**
**Item No. 10**	Disagree	155 (55.2%)	207 (67.4%)	159 (66.5%)	195 (74.7%)	193 (77.2%)	78 (84.8%)	**< 0.001**
**Item No. 11**	Agree	39 (13.9%)	48 (15.6%)	41 (17.2%)	60 (23%)	61 (24.4%)	43 (46.7%)	**< 0.001**
**Item No. 12**	Agree	253 (90%)	283 (92.2%)	221 (92.5%)	239 (91.6%)	226 (90.4%)	87 (94.6%)	0.737
**Item No. 13**	Agree	253 (90%)	285 (92.8%)	219 (91.6%)	234 (89.7%)	230 (92%)	83 (90.2%)	0.756
**Item No. 14**	Disagree	98 (34.9%)	96 (31.3%)	86 (36%)	103 (39.5%)	110 (44%)	43 (46.7%)	**0.013**
**Item No. 15**	Disagree	128 (45.6%)	152 (49.5%)	117 (49%)	144 (55.2%)	140 (56%)	41 (44.6%)	0.084
**Item No. 16**	Agree	19 (6.8%)	23 (7.5%)	20 (8.4%)	35 (13.4%)	36 (14.4%)	4 (4.3%)	**0.002**
**Item No. 17**	Agree	87 (31%)	61 (19.9%)	57 (23.8%)	34 (13%)	36 (14.4%)	12 (13%)	**< 0.001**
**Item No. 18**	Agree	117 (41.6%)	118 (38.4%)	103 (43.1%)	73 (28%)	64 (25.6%)	17 (18.5%)	**< 0.001**
**Item No. 19**	Agree	121 (43.1%)	124 (40.4%)	109 (45.6%)	131 (50.2%)	116 (46.4%)	38 (41.3%)	0.252
**Item No. 20**	Agree	146 (52%)	167 (54.4%)	155 (64.9%)	169 (64.8%)	165 (66%)	64 (69.6%)	**< 0.001**

Chi-squared (χ^2) test was used with a signifcance level (*Sig.*) ≤ 0.05. The significant values are in **bold** font.

Table S2. Lebanese Students' Responses to the Individual HU-DBI Items Stratified by Gender and Clinical Training, 2019/2020, (*n* = 316)

Variable	Outcome	Female (*n* = 210)	Male (*n* = 106)	*Sig.*	Preclinical (*n* = 209)	Clinical (*n* = 107)	*Sig.*
**Item No. 1**	Agree	166 (79%)	82 (77.4%)	0.730	161 (77%)	87 (81.3%)	0.382
**Item No. 2**	Disagree	167 (79.5%)	84 (79.2%)	0.954	156 (74.6%)	95 (88.8%)	**0.003**
**Item No. 3**	Agree	199 (94.8%)	98 (92.5%)	0.415	195 (93.3%)	102 (95.3%)	0.473
**Item No. 4**	Agree	57 (27.1%)	32 (30.2%)	0.570	69 (33%)	20 (18.7%)	**0.007**
**Item No. 5**	Agree	41 (19.5%)	19 (17.9%)	0.732	33 (15.8%)	27 (25.2%)	**0.043**
**Item No. 6**	Disagree	130 (61.9%)	76 (71.7%)	0.084	135 (64.6%)	71 (66.4%)	0.756
**Item No. 7**	Agree	11 (5.2%)	5 (4.7%)	0.842	12 (5.7%)	4 (3.7%)	0.442
**Item No. 8**	Disagree	159 (75.7%)	88 (83%)	0.138	156 (74.6%)	91 (85%)	**0.034**
**Item No. 9**	Agree	157 (74.8%)	85 (80.2%)	0.282	149 (71.3%)	93 (86.9%)	**0.002**
**Item No. 10**	Disagree	152 (72.4%)	84 (79.2%)	0.185	143 (68.4%)	93 (86.9%)	**< 0.001**
**Item No. 11**	Agree	34 (16.2%)	19 (17.9%)	0.697	27 (12.9%)	26 (24.3%)	**0.010**
**Item No. 12**	Agree	193 (91.9%)	95 (89.6%)	0.500	190 (90.9%)	98 (91.6%)	0.841
**Item No. 13**	Agree	192 (91.4%)	99 (93.4%)	0.541	193 (92.3%)	98 (91.6%)	0.814
**Item No. 14**	Disagree	77 (36.7%)	46 (43.4%)	0.247	80 (38.3%)	43 (40.2%)	0.742
**Item No. 15**	Disagree	134 (63.8%)	65 (61.3%)	0.665	126 (60.3%)	73 (68.2%)	0.167
**Item No. 16**	Agree	35 (16.7%)	17 (16%)	0.887	14 (6.7%)	38 (35.5%)	**< 0.001**
**Item No. 17**	Agree	39 (18.6%)	22 (20.8%)	0.642	52 (24.9%)	9 (8.4%)	**< 0.001**

**Item No. 18**	Agree	57 (27.1%)	30 (28.3%)	0.828	69 (33%)	18 (16.8%)	**0.002**
**Item No. 19**	Agree	84 (40%)	38 (35.8%)	0.474	78 (37.3%)	44 (41.1%)	0.511
**Item No. 20**	Agree	126 (60%)	75 (70.8%)	0.061	121 (57.9%)	80 (74.8%)	**0.003**

Chi-squared (χ^2) test was used with a signifcance level (*Sig.*) ≤ 0.05. The significant values are in **bold** font.

Table S3. Syrian Students' Responses to the Individual HU-DBI Items Stratified by Gender and Clinical Training, 2019/2020, (*n* = 561)

Variable	Outcome	Female (*n* = 239)	Male (*n* = 322)	*Sig.*	Preclinical (*n* = 333)	Clinical (*n* = 228)	*Sig.*
**Item No. 1**	Agree	170 (71.1%)	278 (86.3%)	**< 0.001**	269 (80.8%)	179 (78.5%)	0.510
**Item No. 2**	Disagree	160 (66.9%)	216 (67.1%)	0.973	204 (61.3%)	172 (75.4%)	**< 0.001**
**Item No. 3**	Agree	227 (95%)	300 (93.2%)	0.374	311 (93.4%)	216 (94.7%)	0.512
**Item No. 4**	Agree	62 (25.9%)	141 (43.8%)	**< 0.001**	119 (35.7%)	84 (36.8%)	0.789
**Item No. 5**	Agree	88 (36.8%)	82 (25.5%)	**0.004**	99 (29.7%)	71 (31.1%)	0.721
**Item No. 6**	Disagree	159 (66.5%)	189 (58.7%)	0.059	211 (63.4%)	137 (60.1%)	0.432
**Item No. 7**	Agree	21 (8.8%)	51 (15.8%)	**0.014**	43 (12.9%)	29 (12.7%)	0.946
**Item No. 8**	Disagree	172 (72%)	237 (73.6%)	0.666	235 (70.6%)	174 (76.3%)	0.133
**Item No. 9**	Agree	183 (76.6%)	237 (73.6%)	0.423	238 (71.5%)	182 (79.8%)	**0.025**
**Item No. 10**	Disagree	139 (58.2%)	195 (60.6%)	0.567	187 (56.2%)	147 (64.5%)	**0.049**
**Item No. 11**	Agree	40 (16.7%)	66 (20.5%)	0.261	67 (20.1%)	39 (17.1%)	0.382
**Item No. 12**	Agree	216 (90.4%)	279 (86.6%)	0.175	294 (88.3%)	201 (88.2%)	0.962
**Item No. 13**	Agree	210 (87.9%)	291 (90.4%)	0.342	294 (88.3%)	207 (90.8%)	0.346
**Item No. 14**	Disagree	87 (36.4%)	128 (39.8%)	0.420	124 (37.2%)	91 (39.9%)	0.522
**Item No. 15**	Disagree	119 (49.8%)	180 (55.9%)	0.151	171 (51.4%)	128 (56.1%)	0.264
**Item No. 16**	Agree	25 (10.5%)	40 (12.4%)	0.473	36 (10.8%)	29 (12.7%)	0.488
**Item No. 17**	Agree	46 (19.2%)	75 (23.3%)	0.249	84 (25.2%)	37 (16.2%)	**0.011**
**Item No. 18**	Agree	107 (44.8%)	141 (43.8%)	0.817	168 (50.5%)	80 (35.1%)	**< 0.001**
**Item No. 19**	Agree	137 (57.3%)	170 (52.8%)	0.287	168 (50.5%)	139 (61%)	**0.014**
**Item No. 20**	Agree	159 (66.5%)	216 (67.1%)	0.890	215 (64.6%)	160 (70.2%)	0.166

Chi-squared (χ^2) test was used with a signifcance level (*Sig.*) ≤ 0.05. The significant values are in **bold** font.

Table S4. Tunisian Students' Responses to the Individual HU-DBI Items Stratified by Gender and Clinical Training, 2019/2020, (*n* = 553)

Variable	Outcome	Female (*n* = 420)	Male (*n* = 133)	*Sig.*	Preclinical (*n* = 285)	Clinical (*n* = 268)	*Sig.*
**Item No. 1**	Agree	308 (73.3%)	107 (80.5%)	0.098	224 (78.6%)	191 (71.3%)	**0.047**
**Item No. 2**	Disagree	260 (61.9%)	84 (63.2%)	0.795	163 (57.2%)	181 (67.5%)	**0.012**
**Item No. 3**	Agree	404 (96.2%)	124 (93.2%)	0.153	272 (95.4%)	256 (95.5%)	0.962
**Item No. 4**	Agree	142 (33.8%)	59 (44.4%)	**0.027**	95 (33.3%)	106 (39.6%)	0.129
**Item No. 5**	Agree	140 (33.3%)	41 (30.8%)	0.591	78 (27.4%)	103 (38.4%)	**0.006**
**Item No. 6**	Disagree	284 (67.6%)	84 (63.2%)	0.342	175 (61.4%)	193 (72%)	**0.008**
**Item No. 7**	Agree	47 (11.2%)	23 (17.3%)	0.065	34 (11.9%)	36 (13.4%)	0.595
**Item No. 8**	Disagree	308 (73.3%)	95 (71.4%)	0.667	185 (64.9%)	218 (81.3%)	**< 0.001**
**Item No. 9**	Agree	232 (55.2%)	68 (51.1%)	0.407	150 (52.6%)	150 (56%)	0.431
**Item No. 10**	Disagree	327 (77.9%)	90 (67.7%)	**0.017**	191 (67%)	226 (84.3%)	**< 0.001**
**Item No. 11**	Agree	92 (21.9%)	41 (30.8%)	**0.036**	34 (11.9%)	99 (36.9%)	**< 0.001**
**Item No. 12**	Agree	404 (96.2%)	122 (91.7%)	**0.037**	273 (95.8%)	253 (94.4%)	0.450

**Item No. 13**	Agree	388 (92.4%)	124 (93.2%)	0.744	270 (94.7%)	242 (90.3%)	**0.046**
**Item No. 14**	Disagree	152 (36.2%)	46 (34.6%)	0.737	76 (26.7%)	122 (45.5%)	**< 0.001**
**Item No. 15**	Disagree	171 (40.7%)	53 (39.8%)	0.859	100 (35.1%)	124 (46.3%)	**0.007**
**Item No. 16**	Agree	18 (4.3%)	2 (1.5%)	0.134	12 (4.2%)	8 (3%)	0.440
**Item No. 17**	Agree	62 (14.8%)	43 (32.3%)	**< 0.001**	69 (24.2%)	36 (13.4%)	**0.001**
**Item No. 18**	Agree	109 (26%)	48 (36.1%)	**0.024**	101 (35.4%)	56 (20.9%)	**< 0.001**
**Item No. 19**	Agree	166 (39.5%)	44 (33.1%)	0.182	108 (37.9%)	102 (38.1%)	0.968
**Item No. 20**	Agree	223 (53.1%)	67 (50.4%)	0.584	132 (46.3%)	158 (59%)	**0.003**

Chi-squared (χ^2) test was used with a signifcance level (*Sig.*) ≤ 0.05. The significant values are in **bold** font.

Table S5. Pairwise Comprison of Consecutive Academic Levels, Lebanon, 2019/2020, (n = 316)

Pair	Knowledge		Attitudes		Behaviours	
	Mean Rank	*Sig.*	Mean Rank	*Sig.*	Mean Rank	*Sig.*
**1st Year *vs.* 2nd Year**	77.80 / 80.89	0.661	82.55 / 77.01	0.412	72.20 / 85.45	**0.042**
**2nd Year *vs.* 3rd Year**	61.17 / 83.71	**0.001**	66.48 / 74.65	0.212	70.02 / 68.61	0.817
**3rd Year *vs.* 4th Year**	44.19 / 44.93	0.887	42.65 / 47.05	0.398	42.83 / 46.80	0.422
**4th Year *vs.* 5th Year**	54.04 / 53.98	0.992	58.45 / 54.65	0.249	49.85 / 56.19	0.269

Mann-Whitney (U) test was used with a signifcance level (*Sig.*) ≤ 0.05. The significant values are in **bold** font.

Table S6. Pairwise Comprison of Consecutive Academic Levels, Syria, 2019/2020, (n = 561)

Pair	Knowledge		Attitudes		Behaviours	
	Mean Rank	*Sig.*	Mean Rank	*Sig.*	Mean Rank	*Sig.*
**1st Year *vs.* 2nd Year**	108.79 / 108.22	0.946	107.75 / 109.23	0.853	105.40 / 111.54	0.429
**2nd Year *vs.* 3rd Year**	117.24 / 110.01	0.392	123.19 / 104.47	**0.022**	106.80 / 119.74	0.102
**3rd Year *vs.* 4th Year**	109.97 / 136.63	**0.003**	118.10 / 129.31	0.187	124.42 / 123.62	0.922
**4th Year *vs.* 5th Year**	112.08 / 117.71	0.509	113.56 / 115.74	0.790	115.96 / 112.56	0.671

Mann-Whitney (U) test was used with a signifcance level (*Sig.*) ≤ 0.05. The significant values are in **bold** font.

Table S7. Pairwise Comprison of Consecutive Academic Levels, Tunisia, 2019/2020, (n = 553)

Pair	Knowledge		Attitudes		Behaviours	
	Mean Rank	*Sig.*	Mean Rank	*Sig.*	Mean Rank	*Sig.*
**1st Year *vs.* 2nd Year**	95.81 / 118.35	**0.006**	106.95 / 108.01	0.891	109.37 / 105.76	0.643
**2nd Year *vs.* 3rd Year**	91.82 / 90.99	0.915	90.76 / 92.66	0.791	88.60 / 96.04	0.308
**3rd Year *vs.* 4th Year**	74.89 / 89.12	**0.050**	70.51 / 92.43	**0.002**	83.64 / 82.52	0.869
**4th Year *vs.* 5th Year**	89.88 / 86.92	0.690	84.12 / 93.52	0.193	87.85 / 89.25	0.842
**5th Year *vs.* 6th Year**	82.91 / 91.59	0.235	85.57 / 89.22	0.613	85.71 / 89.10	0.623

Mann-Whitney (U) test was used with a signifcance level (*Sig.*) ≤ 0.05. The significant values are in **bold** font.

Table S8. Knowledge, Attitudes, Behaviours and Total HU-DBI Score of the Lebanese Dental Students, 2019/2020, (n = 316)

Variable	Outcome	Knowledge (0 – 5)	*Sig.*	Attitudes (0 – 3)	*Sig.*	Behaviours (0 – 4)	*Sig.*	HU-DBI (0 – 12)	*Sig.*
**Sex**	Female	3.31 ± 1.19	0.668	1.15 ± 0.80	0.088	2.10 ± 0.74	0.677	6.57 ± 1.92	0.212
	Male	3.39 ± 1.09		1.33 ± 0.80		2.16 ± 0.71		6.88 ± 1.61	
**Academic Year**	1st Year	2.92 ± 1.26	**< 0.001**	1.15 ± 0.71	0.258	1.87 ± 0.83	**0.001**	5.94 ± 1.92	**< 0.001**
	2nd Year	3.05 ± 1.09		1.08 ± 0.78		2.10 ± 0.67		6.23 ± 1.46	
	3rd Year	3.67 ± 1.05		1.29 ± 0.90		2.08 ± 0.63		7.04 ± 1.72	
	4th Year	3.73 ± 0.99		1.43 ± 0.90		2.24 ± 0.83		7.41 ± 1.79	

	5th Year	3.69 ± 1.06		1.24 ± 0.79		2.37 ± 0.64		7.30 ± 1.85	
**Clinical Training**	Preclinical	3.15 ± 1.18	**< 0.001**	1.16 ± 0.79	0.130	2.02 ± 0.72	**< 0.001**	6.33 ± 1.74	**< 0.001**
	Clinical	3.70 ± 1.03		1.31 ± 0.83		2.33 ± 0.71		7.34 ± 1.82	
**Tobacco Smoking**	Yes	3.35 ± 1.10	0.951	1.19 ± 0.77	0.726	2.12 ± 0.74	0.870	6.65 ± 1.70	0.647
	No	3.34 ± 1.17		1.21 ± 0.82		2.13 ± 0.73		6.68 ± 1.86	
**Internet Addiction**	Yes	3.32 ± 1.18	0.472	1.21 ± 0.79	0.711	2.12 ± 0.74	0.529	6.65 ± 1.81	0.424
	No	3.47 ± 0.97		1.19 ± 0.92		2.17 ± 0.70		6.83 ± 1.94	

Mann-Whitney (*U*) and Kruskal-Wallis (*H*) tests were used with a signifcance level (*Sig.*) ≤ 0.05. The significant values are in **bold** font.

Table S9. Knowledge, Attitudes, Behaviours and Total HU-DBI Score of the Syrian Dental Students, 2019/2020, (*n* = 561)

Variable	Outcome	Knowledge (0 – 5)	*Sig.*	Attitudes (0 – 3)	*Sig.*	Behaviours (0 – 4)	*Sig.*	HU-DBI (0 – 12)	*Sig.*
**Sex**	Female	3.04 ± 1.31	0.769	1.20 ± 0.82	0.834	2.03 ± 0.72	**0.035**	6.27 ± 1.95	0.418
	Male	3.10 ± 1.21		1.19 ± 0.89		2.16 ± 0.80		6.45 ± 1.73	
**Academic Year**	1st Year	2.93 ± 1.40	**0.002**	1.28 ± 0.88	0.131	1.95 ± 0.83	0.064	6.17 ± 1.91	**0.019**
	2nd Year	2.94 ± 1.23		1.32 ± 0.93		2.04 ± 0.76		6.29 ± 1.95	
	3rd Year	2.83 ± 1.20		1.03 ± 0.86		2.19 ± 0.78		6.05 ± 1.80	
	4th Year	3.28 ± 1.21		1.15 ± 0.79		2.21 ± 0.70		6.64 ± 1.69	
	5th Year	3.41 ± 1.13		1.19 ± 0.85		2.13 ± 0.77		6.73 ± 1.73	
**Clinical Training**	Preclinical	2.90 ± 1.28	**< 0.001**	1.21 ± 0.90	0.807	2.06 ± 0.79	0.102	6.17 ± 1.88	**0.001**
	Clinical	3.33 ± 1.18		1.17 ± 0.81		2.18 ± 0.73		6.68 ± 1.71	
**Tobacco Smoking**	Yes	3.00 ± 1.22	0.241	1.12 ± 0.88	0.123	2.18 ± 0.80	0.313	6.29 ± 1.75	0.267
	No	3.11 ± 1.27		1.23 ± 0.85		2.08 ± 0.75		6.41 ± 1.86	
**Alcohol Drinking**	Yes	2.93 ± 1.37	0.503	1.00 ± 0.86	0.130	2.07 ± 0.80	0.645	6.00 ± 1.77	0.199
	No	3.09 ± 1.25		1.21 ± 0.85		2.11 ± 0.77		6.41 ± 1.83	
**Internet Addiction**	Yes	3.00 ± 1.25	**0.007**	1.18 ± 0.86	0.465	2.09 ± 0.79	0.432	6.28 ± 1.85	**0.014**
	No	3.34 ± 1.25		1.23 ± 0.87		2.18 ± 0.69		6.76 ± 1.67	

Mann-Whitney (*U*) and Kruskal-Wallis (*H*) tests were used with a signifcance level (*Sig.*) ≤ 0.05. The significant values are in **bold** font.

Table S10. Knowledge, Attitudes, Behaviours and Total HU-DBI Score of the Tunisian Dental Students, 2019/2020, (*n* = 553)

Variable	Outcome	Knowledge (0 – 5)	*Sig.*	Attitudes (0 – 3)	*Sig.*	Behaviours (0 – 4)	*Sig.*	HU-DBI (0 – 12)	*Sig.*
**Sex**	Female	2.93 ± 1.24	0.233	1.26 ± 0.86	0.756	1.90 ± 0.73	0.844	5.92 ± 1.77	0.432
	Male	2.75 ± 1.19		1.29 ± 0.83		1.89 ± 0.73		6.09 ± 1.84	
**Academic Year**	1st Year	2.33 ± 1.33	**< 0.001**	0.99 ± 0.79	**< 0.001**	1.86 ± 0.78	0.680	5.18 ± 1.89	**< 0.001**
	2nd Year	2.79 ± 1.19		0.99 ± 0.68		1.82 ± 0.73		5.60 ± 1.78	
	3rd Year	2.77 ± 1.26		1.03 ± 0.77		1.92 ± 0.67		5.72 ± 1.84	
	4th Year	3.16 ± 1.08		1.41 ± 0.75		1.90 ± 0.73		6.48 ± 1.70	
	5th Year	3.06 ± 1.15		1.57 ± 0.92		1.91 ± 0.72		6.55 ± 1.57	
	6th Year	3.29 ± 0.94		1.65 ± 0.83		1.97 ± 0.72		6.91 ± 1.53	
**Clinical Training**	Preclinical	2.62 ± 1.27	**< 0.001**	1.00 ± 0.74	**< 0.001**	1.86 ± 0.73	0.203	5.48 ± 1.84	**< 0.001**
	Clinical	3.18 ± 1.06		1.54 ± 0.84		1.93 ± 0.72		6.65 ± 1.61	
**Tobacco Smoking**	Yes	2.62 ± 1.30	**0.013**	1.09 ± 0.77	**0.018**	1.85 ± 0.75	0.403	5.56 ± 1.81	**0.001**
	No	2.95 ± 1.18		1.30 ± 0.84		1.90 ± 0.72		6.16 ± 1.82	
**Alcohol Drinking**	Yes	2.54 ± 1.41	0.082	1.24 ± 0.72	0.969	1.89 ± 0.74	0.945	5.68 ± 1.90	0.270
	No	2.91 ± 1.19		1.27 ± 0.84		1.89 ± 0.73		6.07 ± 1.82	

**Internet Addiction**	Yes	2.87 ± 1.22	0.142	1.27 ± 0.83	0.470	1.90 ± 0.73	0.363	6.04 ± 1.83	0.967
	No	3.18 ± 1.00		1.18 ± 0.90		1.79 ± 0.64		6.15 ± 1.73	

Mann-Whitney (*U*) and Kruskal-Wallis (*H*) tests were used with a signifcance level (*Sig.*) ≤ 0.05. The significant values are in **bold** font.

Table S11. HU-DBI Score of Dental Students in the Arab League Member States, 2006 – 2020, (*n* = 6941)

**Study** Author, Year	**Item 2** *Disagree*	**Item 4** *Agree*	**Item 6** *Disagree*	**Item 8** *Disagree*	**Item 9** *Agree*	**Item 10** *Disagree*	**Item 11** *Agree*	**Item 12** *Agree*	**Item 14** *Disagree*	**Item 15** *Disagree*	**Item 16** *Agree*	**Item 19** *Agree*	**Total** Score
Al-wesabi *et al.*, 2015 [58]	70.2%	37.4%	85.7%	77.5%	65.6%	64.8%	21.2%	82.3%	39.9%	43.9%	6.0%	39.9%	6.77 ± 1.73
Abu Alregal *et al.*, 2016 [63]	75.9%	31.5%	84.8%	79.8%	69.2%	65.7%	22.3%	80.0%	39.2%	41.7%	6.2%	36.8%	6.33 ± 1.66
Al-wahadni et al., 2004 [64]	87.9%	23%	46.6%	70.1%	70.1%	87.9%	7%	77.1%	51.4%	57%	18%	42%	6.38 ± 1.72
Al-omiri *et al.*, 2010 [65]	73.8%	26.9%	56.4%	72.2%	78.1%	66.7%	11.4%	89.0%	47.4%	39.8%	15.5%	40.2%	5.2 ± 1.57
Ali D., 2015 [66]	79%	24%	59%	82%	78%	62%	11%	12%	40%	47%	19%	20%	5.74 ± 0.23
Riad *et al.* 2020 [Lebanon]	79.4%	28.2%	65.2%	78.2%	76.6%	74.7%	16.8%	91.1%	38.9%	63%	16.5%	38.6%	6.67 ± 1.83
Kateeb E., 2006 [57]	68.1%	37.7%	45.4%	60.8%	72.3%	73.1%	16.9%	83.5%	40%	49.6%	16.9%	48.5%	6.13 ± 1.31
Baseer *et al.*, 2011 [68]	63.2%	24.2%	66.1%	68.9%	69.8%	54.4%	29.6%	78.6%	46.7%	49.9%	44.7%	58.1%	6.54 ± 1.07
Kumar et al., 2011 [69]	61.5%	34.5%	65.2%	63.9%	73.1%	61.5%	42.3%	88.4%	34.6%	57.6%	57.5%	61.5%	6.65 ± 1.08
Moheet *et al.*, 2013 [70]	75%	36.6%	64.2%	72.3%	79.5%	64.2%	13.4%	67.9%	43.75%	55.4%	26.8%	45.5%	6.45 ± 1.33
Khalid *et al.*, 2011 [71]	82.1%	40.3%	64.1%	63.0%	88.5%	56.5%	9.1%	70.9%	39.1%	34.5%	7.2%	69.0%	6.24 ± 1.64
Riad *et al.*, 2020 [Syria]	67%	36.2%	62%	72.9%	74.9%	59.5%	18.9%	88.2%	38.3%	53.3%	11.6%	54.7%	6.38 ± 1.83
Riad *et al.*, 2020 [Tunisia]	62.2%	36.3%	66.5%	72.9%	54.2%	75.4%	24.1%	95.1%	35.8%	40.5%	3.6%	38%	6.05 ± 1.83
Kawas *et al.*, 2009 [72]	75%	24%	73%	73%	55%	88%	9%	77%	47%	80%	17%	59%	9.45 ± 1.48
Hashim *et al.*, 2012 [73]	79.6%	26.9%	61.6%	80.6%	79.6%	72.8%	12.5%	84.2%	49.8%	47.3%	21.1%	43.4%	6.59 ± 1.89
Rahman *et al.*, 2013 [74]	71.4%	10.4%	91.6%	75.5%	47.6%	99.1%	1.7%	69.1%	62.4%	80.2%	91.1%	31.5%	7.32 ± 1.53
Halboub *et al.* 2015 [19]	46.9%	76.9%	82.9%	29.0%	69.6%	94.1%	17.7%	76.1%	22.3%	68.9%	0%	26.1%	5.06 ± 1.63
